# European Correspondence

**Published:** 1876-06

**Authors:** 


European Correspondents.—We are happy to be able to state that we have perfected arrangements by which we will be able to present one or more letters from the medical centres of Europe in each succeeding number of the Journal for the next two years.

Dr. W. S. Kendrick, who is connected with the editorial department of the Journal, and Dr. H. F. Scott, Demonstrator of Anatomy in the Atlanta Medical College, sailed for Europe the last of May, and will, during their stay in Europe, contribute regularly to the Journal. Dr. R. J. Nunn, a prominent physician of Savannah, Georgia, is now in the North, and will spend a few weeks in New York and Philadelphia, and has promised us letters from those points. He will sail for Europe in August or September, where he will remain for two or three years, and has promised us one or more letters for each number of the Journal during his stay.

